# Supplementary material for: Membrane-assisted assembly and selective secretory autophagy of enteroviruses
Source: Nat Commun. 2022 Oct 10;13:5986. doi: 10.1038/s41467-022-33483-7 (PMC9550805; doi:10.1038/s41467-022-33483-7)
Supplement: Supplementary file 1 — Supplementary Information File [file 41467_2022_33483_MOESM1_ESM.pdf]

SUPPLEMENTARY INFORMATION

Membrane-assisted assembly and selective secretory autophagy of enteroviruses

Selma Dahmane[1,2,3], Adeline Kerviel[4], Dustin R. Morado[5], Kasturika Shankar[1,2,3], Björn Ahlman[1,2,3], Michael Lazarou[6], Nihal Altan-Bonnet[4], Lars-Anders Carlson[1,2,3,*]

[1]Department of Medical Biochemistry and Biophysics, Umeå University, Umeå, Sweden.

[2]Wallenberg Centre for Molecular Medicine, Umeå University, Umeå, Sweden.

[3]Molecular Infection Medicine Sweden, Umeå University, Umeå, Sweden.

[4]Laboratory of Host-Pathogen Dynamics, National Heart Lung and Blood Institute, National Institutes of Health, Bethesda, MD, USA.

[5]Department of Biochemistry and Biophysics, Science for Life Laboratory, Stockholm University, Stockholm, Sweden.

[6]Department of Biochemistry and Molecular Biology, Biomedicine Discovery Institute, Monash University, Melbourne, Australia.

*correspondence: lars-anders.carlson@umu.se

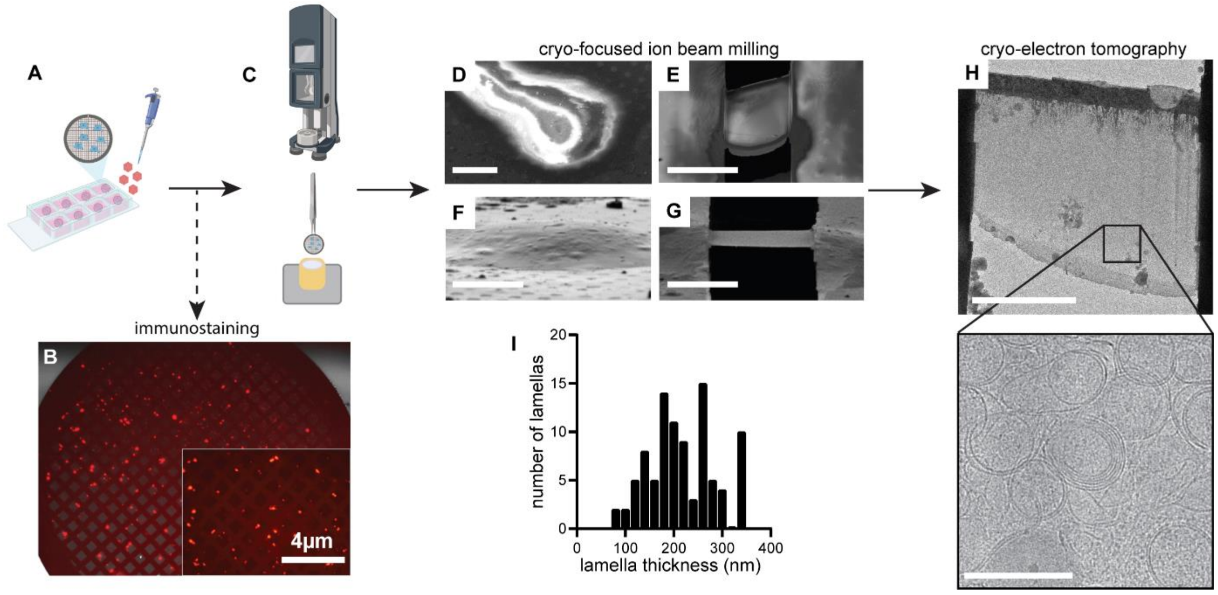

Supplementary figure 1: Experimental workflow for cryo-electron tomography. (A) HeLa cells were cultured on gold EM grids in chambered slides and infected with poliovirus at MOI 5. Created with BioRender.com. (B) Immunostaining against the viral capsid protein VP1, with a secondary antibody conjugated to Alexa584 (red), confirmed PV infection of virtually all cells growing on EM grids. (C) Unfixed and unlabeled infected cells were vitrified by plunge freezing. (D-E) Scanning electron microscopy and (F-G) ion beam images before and after the FIB-milling process of a PV-infected cell, resulting in thin lamella (here seen at a slight angle). (H) Low magnification transmission electron microscopy (TEM) image of the thin transparent cryo-lamella, where areas containing DMVs were abundant (zoomed box). (I) Thickness distribution of cryo-lamellas obtained with TEM. Average thickness is 217 ± 69 nm; n = 93 lamellas. Scale bars: (F-G) 5 µm, (H) 200 nm.

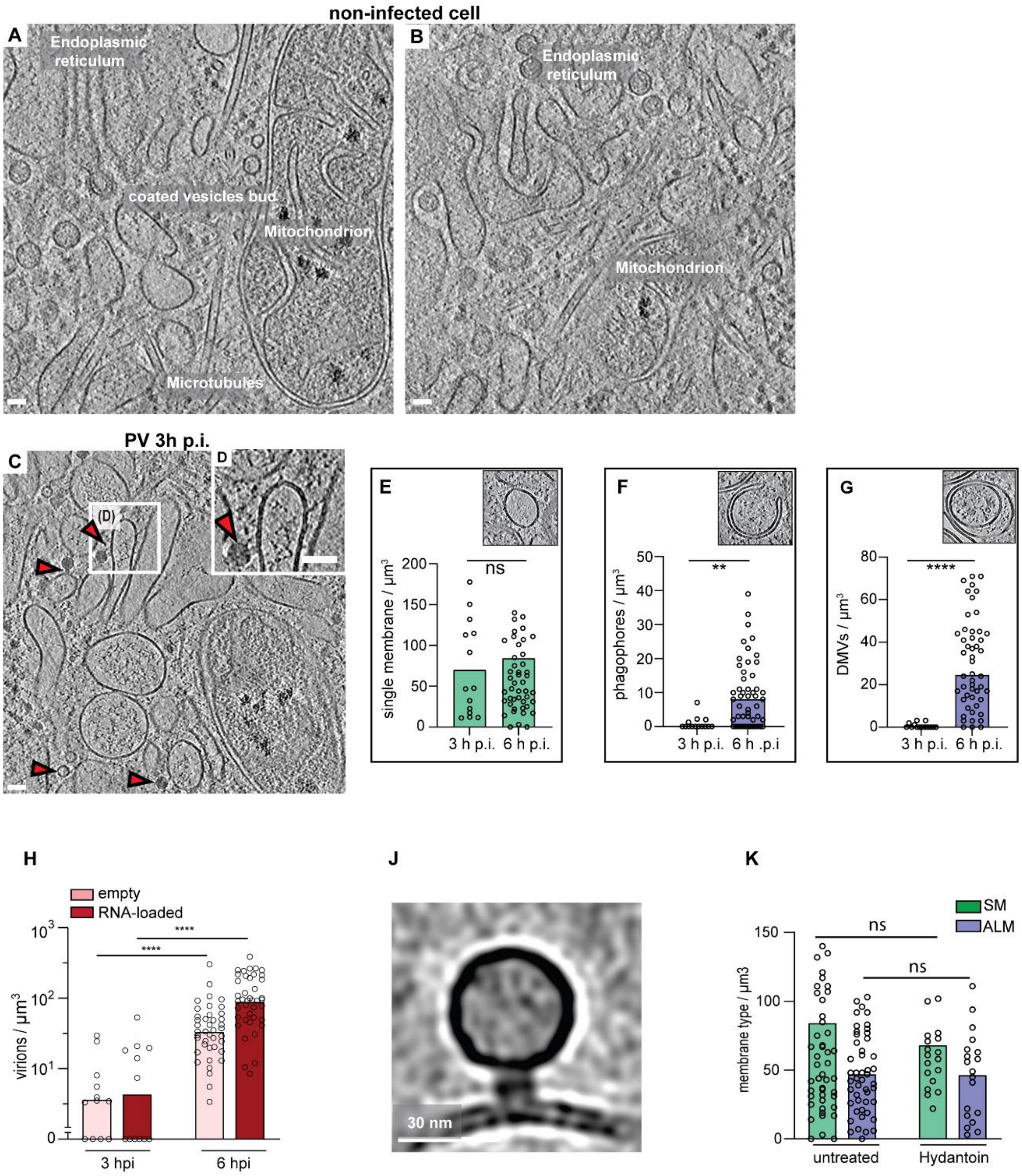

Supplementary figure 2: Cryo-ET of poliovirus-infected cell at 3 h p.i. (A-B) Slices through a tomogram of an uninfected HeLa cell showing several cytoplasmic features and organelles as labeled. (C) Slice through a cryo-electron tomogram of a lamella milled through a PV-infected cell at 3 h p.i., revealing PV-induced SM proliferation. (D) Magnified view of the white box in (C) showing a tethered virion to SM. (C-D) Red arrowheads indicate viral particles. Scale bars: 50 nm. (E-G) Scatter plots with bars representing the mean concentration of SM structures (E) phagophore-like structures (F), and DMVs (G), observed in (n) cryo-tomograms of 3 h p.i. (n = 14) and 6 h p.i (n = 50). Insets: slices through different tomograms showing the type of membrane for each graph. (H) Concentration of intracellular empty capsids and RNA-loaded virions as measured by template matching in (n) tomograms at 3 h p.i. (n = 14) and 6

h p.i.,(n = 50). (J) Central slice through the unsymmetrized subtomogram average of the tethered viral capsid, low-pass filtered to its Gold-standard resolution 39 Å (corresponding to Fig 1F). (K) Scatter plot with bars representing the mean concentration of SM and ALM measured in (n) tomograms of Hydantoin-treated cells (n = 19) at 6 h p.i. compared to untreated cells (n = 50). Each dot corresponds to one tomogram, bars represent the averages (see also Supplementary table 2). Statistical significance by unpaired two-tailed Student's t test: (E) p = 0,0687, (F) p = 0,0002, (G-H) p<0,0001, (K) for SM p = 0,47; ALM p = 0,87.

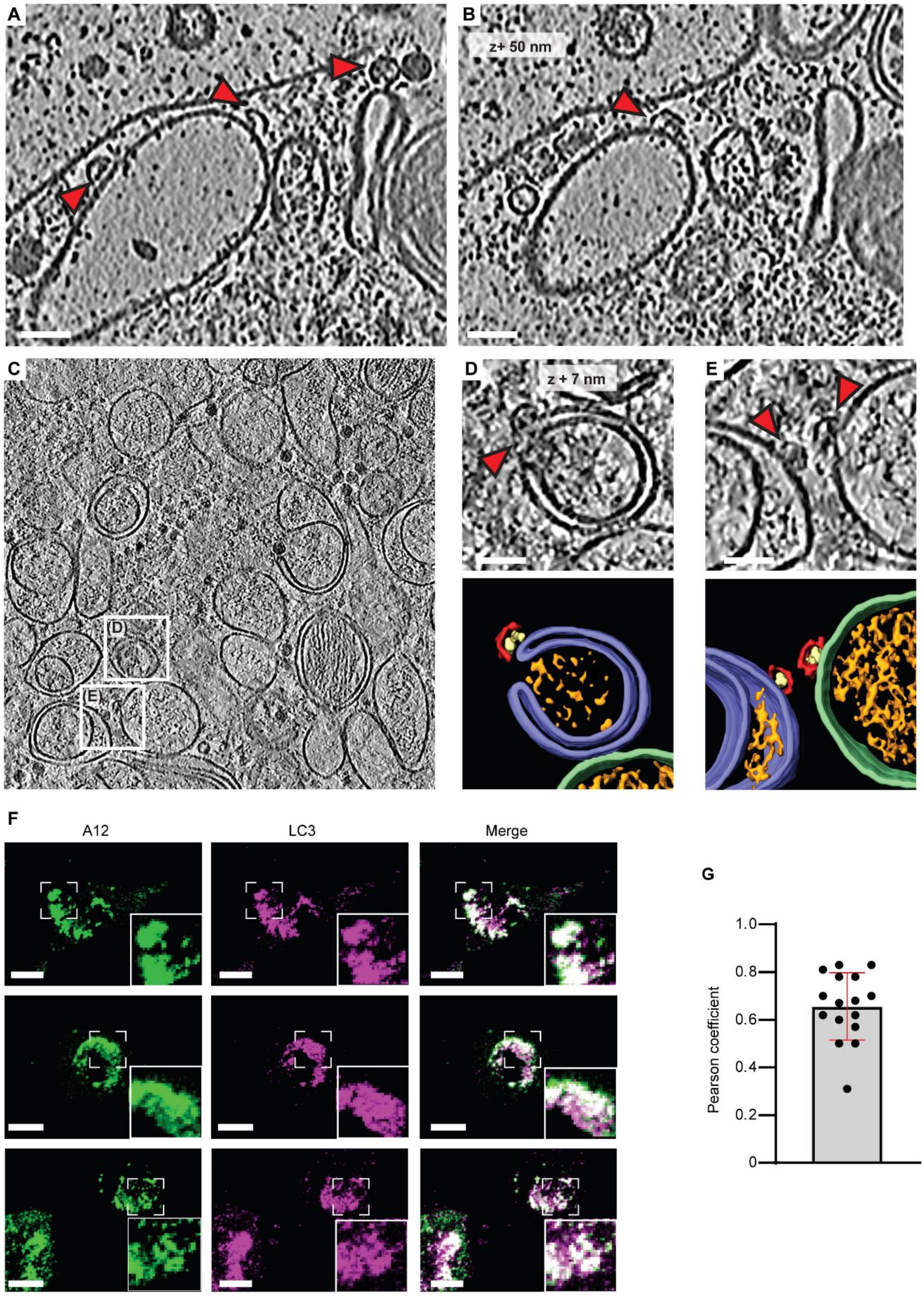

Supplementary figure 3: capsid intermediates bound to SM and ALM. (A-B) Slices of PV-infected cell at 6 h p.i. cryo-tomogram corresponding to the segmentation in Figure 2A, showing capsid intermediates bound to SM (red arrowheads). (C) Cryo-electron tomogram of a PV-infected cell at 6 h p.i. White boxes indicate areas with capsid assembly intermediates.

(D-E) Magnified views of boxes in (C) with capsid intermediates indicated by red arrowheads, and their corresponding 3D segmentations showing capsid assembly intermediates (red) containing luminal densities (yellow), associated with autophagy-like membranes (ALMs, purple) and a single-membrane vesicle (SM, green). (F) HeLa cells infected with PV for 6 h and stained for assembled provirions and mature viruses using the A12 antibody (green) and LC3B (magenta). Areas of colocalization appear in white. (G) Quantification of assembled virions and LC3B colocalization was assessed with ImageJ and expressed as Pearson's correlation coefficient Error bar represent mean and standard deviation (0,66 ± 0,14) for n = 16 cells. Scale bars: (A-D) 50 nm, (F) 10 µm.

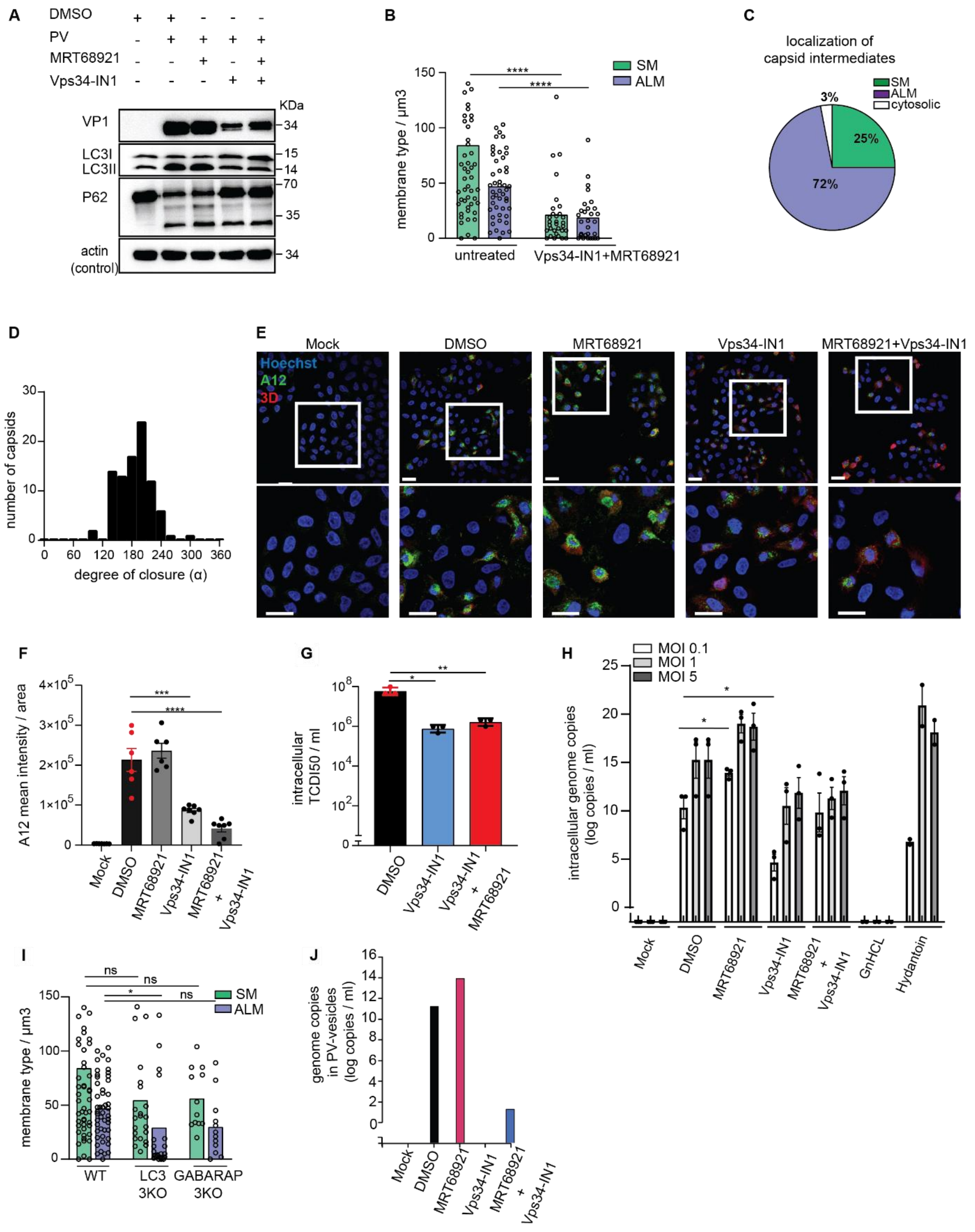

Supplementary figure 4: Characterization of PV-infection in Vps34-IN1 treated cells. (A) LC3B lipidation in PV-infected cells at 6 h p.i. treated or not with the autophagy inhibitors MRT68921 (1 µM) and/or Vps34-IN1 (5 µM). Also shown is the autophagy receptor p62/SQSTM1 which is characteristically cleaved during PV infection. Cell lysates were immunoblotted against the indicated proteins. LC3II, lipidated form of LC3B protein. (B) Concentration of SMs and ALMs measured in (n) tomograms of untreated (n = 50) and Vps34-

IN1 + MRT68921 (n = 31) treated cells at 6 h p.i. Statistical significance by unpaired two-tailed Student’s t test; ***p=0,0005, ****p<0.0001. (C) Percentage of capsid assembly intermediates found on SMs, ALMs or not associated with membranes, as counted in 17 cryo-tomograms of Vps34-IN1 treated cells at 6 h p.i. (D) Distribution of capsid intermediate closures observed in tomograms of Vps34-IN1 + MRT68921-treated cells at 6 h p.i. The average closure is 186° (SD=35°, N=90). (E) Immunofluorescence assay of PV-infected cells in the presence or absence of autophagy inhibitors MRT68921 and Vps34-IN1 at 6 h p.i. Assembled provirions and mature viruses were detected using the A12 antibody (green) and the expression of the non-structural protein $3D^{pol}$ was verified concomitantly (red). (F) Quantification of the A12 mean fluorescence intensities obtained from immunofluorescence images illustrated in (B) measured in 6 and 7 independent experiments. Bars represent the means ± SD. Statistical significance by unpaired two-tailed Student’s t test; ***p=0,0007, - ****p<0.0001. (G) Intracellular PV titers measured at 6 h p.i. in the presence of Vps34-IN1 and MRT68921. Bars represent the means of biological triplicates ± SEM. Statistical significance by unpaired two-tailed Student’s t test; *p=0,01, **p=0,05. (H) Intracellular viral RNA copies measured at 6 h p.i. in cells infected with PV with MOI 5, 1 and 0.1; and treated with DMSO, Vps34-IN1, Vps34-IN1 + MRT68921, guanidine HCl (GnHCL) or Hydantoin. Bars represent the means of biological triplicates and duplicates (GnHCL and Hydantoin) ± SEM. No statistical significance was observed in the majority of conditions except MOI=0.1 treatment with MRT68921 (increase, *p=0,01) and VPS34-IN1 (decrease, *p=0,04). Statistical significance derived by unpaired two-tailed Student’s t test. (I) Concentration of SMs and ALMs measured in (n) tomograms of WT (n = 50), LC3 (n = 23) and GABARAP 3KO cells (n = 13) at 6 h p.i. Statistical significance by unpaired two-tailed Student’s t test, *p=0,04. (B-I) Each dot is one tomogram. The bars represent the average. Numerical source data are presented in Supplementary Table 2. Scale bars: 20 µm. (K) Extracellular viral RNA copies measured at 6 h p.i. in extracellular vesicles harvested from cells infected with PV at MOI 5 and treated with DMSO, MRT68921 alone and/or with Vps34-IN1.

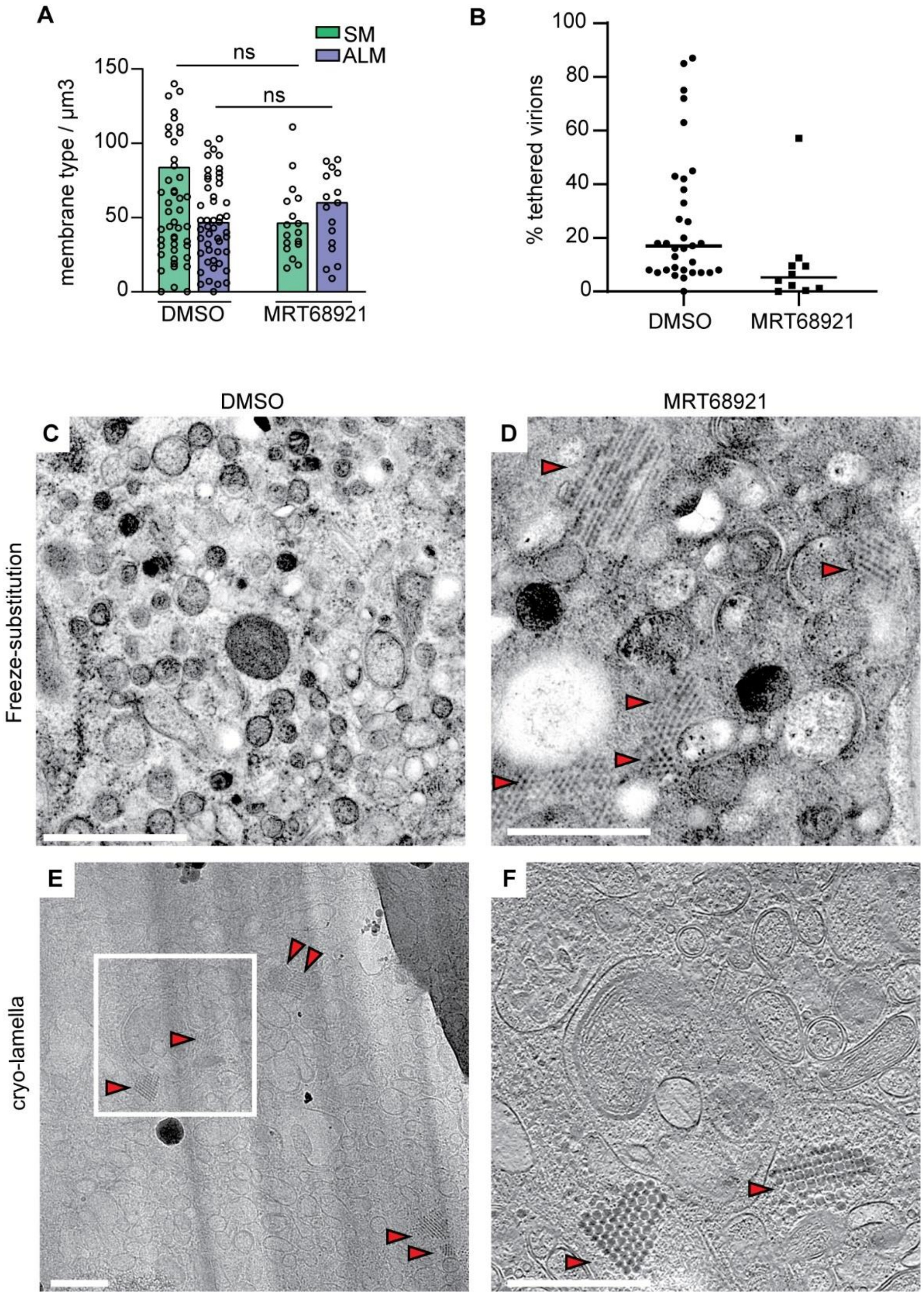

Supplementary figure 5: Intracellular virus array formation in infected cells treated with MRT68921. (A) Concentration of SMs vs. ALMs observed in (n) cryo-tomograms of MRT68921 treated cells (n = 50) compared to untreated cells (n = 17) at 6 h p.i. Each dot corresponds to one tomogram analyzed and bars represent the mean (see also Supplementary table 2). (B) Percentage of tethered virions measured in (n) tomograms of untreated (n = 33) and MRT68921-treated (n = 10) cells at 6h p.i. Bars represent the mean. (C-D) Electron micrographs of thin sections of freeze-substituted PV-infected cells in the absence (C) and

presence (D) of MRT68921 at 6 h p.i., indicating the formation of virus arrays (red arrowheads) when cells are treated with MRT68921. (E) Low magnification image of cryo-lamella milled through MRT68921 treated cell at 6 h p.i. is included for comparison with (D), in which virus arrays are already identified (red arrowhead). (F) Corresponding slice through the tomogram reconstructed from tilt-series collected on the indicated region (white frames in (C)). Scale bars: 500 nm.

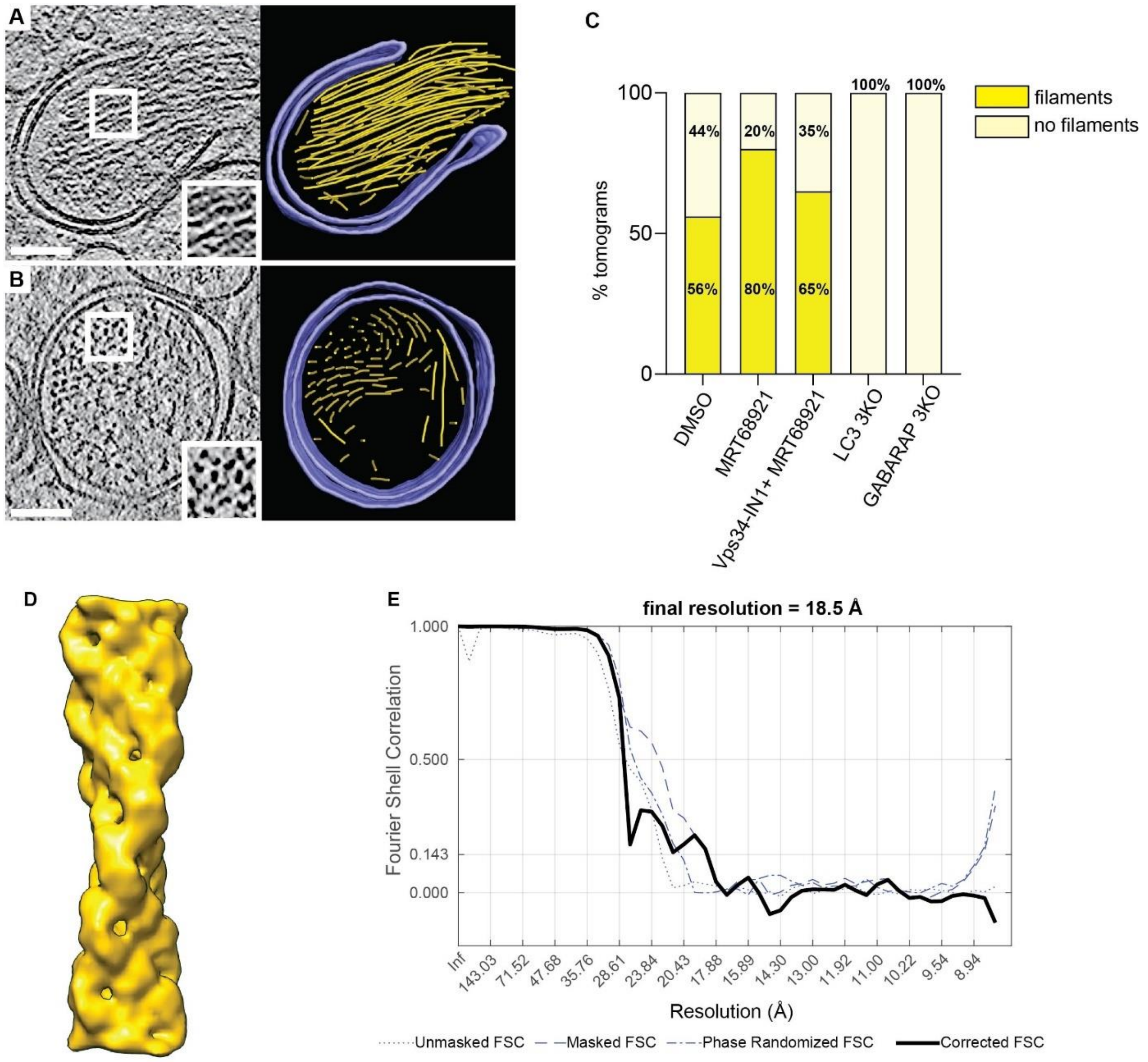

Supplementary figure 6: ALM-associated protein filament. (A-B) Slices through tomograms of PV-infected cells at 6 h p.i and their corresponding 3D segmentation showing large bundles of the filament (yellow) filling the interior of a phagophore-like structure (A) and double-membrane vesicle (B) (purple). Zoomed regions (white boxes) highlight the helical twist (A) and bundle formation (B) of the filaments. Scale bars: 100 nm. (C) Percentage of tomograms containing ALM-associated protein filament bundles in DMSO treated WT cells (n=34), in MRT68921 treated WT cells (n=10) and in MRT68921 + Vps34-IN1 treated WT cells (n=20), compared to LC3 (n=17) and GABARAP (N=13) 3KO cells. (D) Subtomogram average of the filaments at 18.5 Å resolution. (E) Fourier shell correlation curves for unmasked, masked, and phase-randomized (beyond 31 Å) half-sets. The corrected curve, equaling 0.143 at 18.5 Å resolution, is based on Chen *et al*[59] as implemented in subTOM.

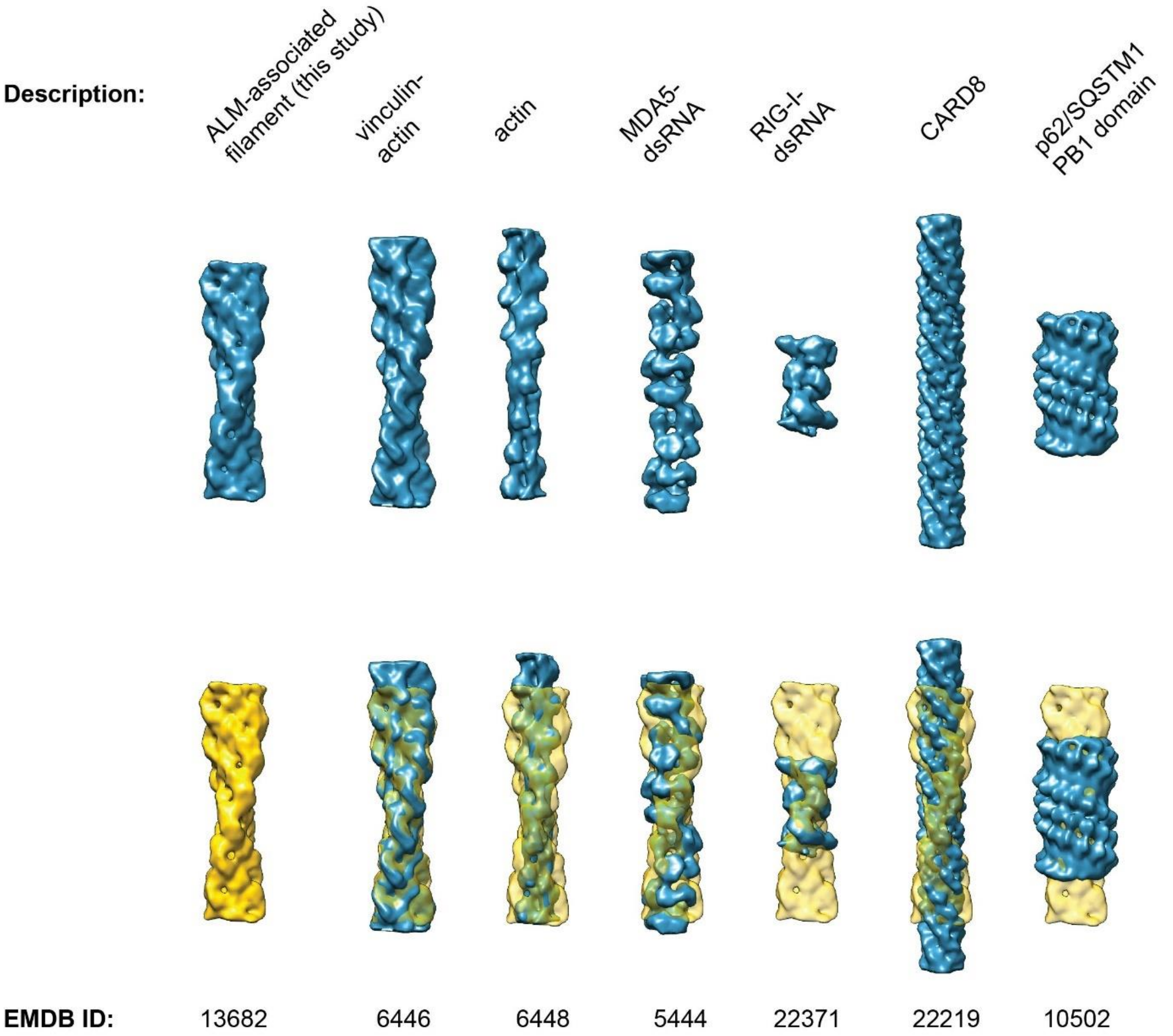

Supplementary figure 7: Comparison of the ALM-associated filament with a selection of known filament structures. Top row: isosurface representations of the subtomogram average of the ALM-associated protein filament from this study, and one representative of each of several classes of cellular protein filaments with known structure. In the lower row, the ALM-associated filament is shown in yellow, and semitransparent yellow when fitted to each of the other filaments using UCSF Chimera's Fit in Map function[45]. Prior to comparison, all filaments were resampled to the same voxel size and filtered to 19 Å resolution. EMDB identifiers are indicated below each volume.

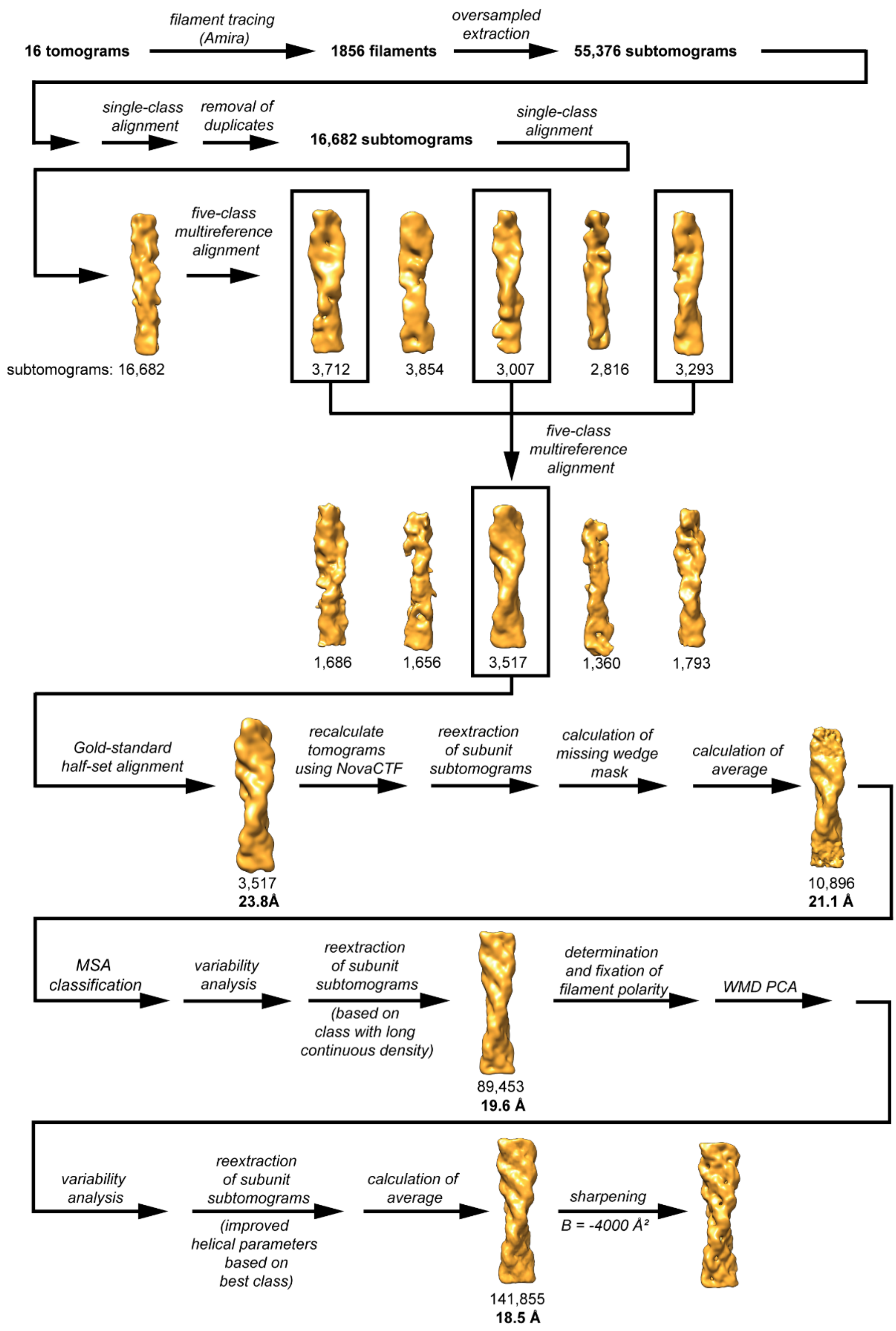

Supplementary figure 8: Schematic of the workflow for subtomogram averaging of the tethered and non-tethered virions. The schematic illustrates the major steps of the data processing starting at particle picking in the tomograms, and extraction of subtomograms, through the several steps of classification and averaging.

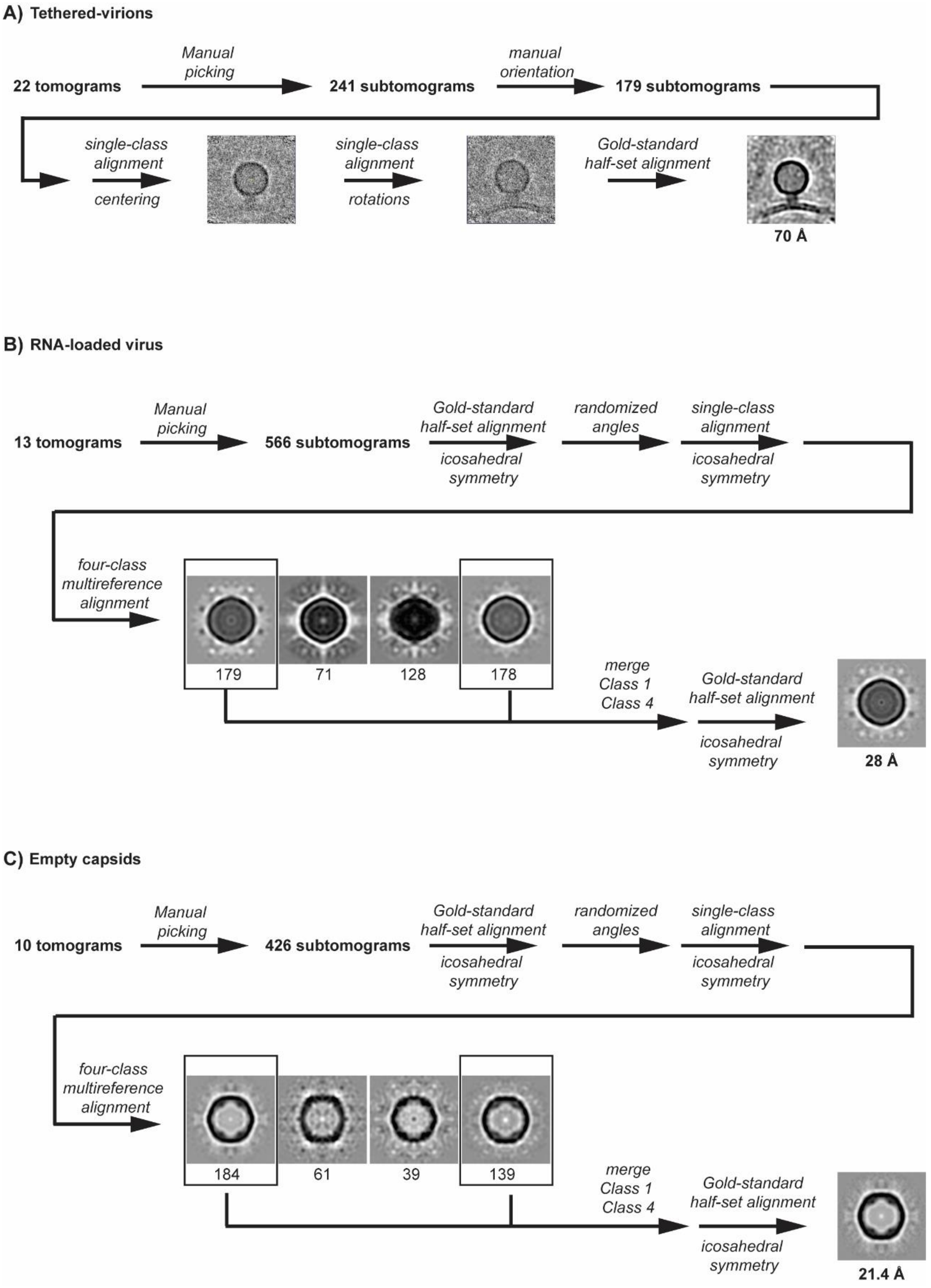

Supplementary figure 9: Schematic of the workflow for subtomogram averaging of the ALM-associated filament. The schematic illustrates the major steps of the data processing starting at filament tracing in the tomograms, and extraction of subtomograms along the filament axis, through the several steps of classification, reextraction and averaging.

Supplementary table 1: Data collection and parameter for Cryo-electron tomography of poliovirus replication and assembly sites.

Data collection	
microscope	Titan Krios G2
Voltage (KeV)	300
Camera	Gatan K2
Magnification	33,000
Energy filter	yes, BioQuantum
Slit width (eV)	20
Pixel size in super-resolution mode (Å)	2.18
Defocus range (µm)	-3 to -5
Tilt range (°)	± 50 to ± 60
Total dose (e−/Å^2)	100 to 130
Tomograms acquired	168

Supplementary table 2: Concentration of membrane structures and virions (Mean ± SD).

	N tomograms	Figures	SM / µm^3	ALM / µm^3	RNA-loaded virions / µm^3	Empty Capsids / µm^3
PV 3 hpi	14	Supplementary figure 1C	70,04 ± 58,08	1,21 ± 1,31	10,11 ± 14,74	6,26 ± 9,05
PV 6 hpi	50	1A-D, 2B-E, 5A-E, 5H-M, Supplementary figure 1D-H, 2A-E, 6A-B	84,12 ± 92,66	45,08 ± 29,23	106,11 ± 87,66	40,47 ± 48,91
MRT68921	17	4A, 4D-G Supplementary figure 5C-F	48,11 ± 25	60,47 ± 38,56	546,23 ± 737,61	127,53 ± 114,82
VPS34-IN1 + MRT68921	31	3A-D	21,42 ± 27,85	17,45 ± 20,69	2,21 ± 3,12	3,78 ± 5,3
Hydantoin	19	1I	68,16 ± 44,88	29,17 ± 49,64	50,55 ± 54,84	152,83 ± 88,98
3KO LC3	23	3H-I	54,52 ± 47,52	28,01 ± 27,21	39,61 ± 112,5	19,19 ± 40,92
3KO GABARAP	13	3J	53,69 ± 28,1	46,37 ± 31,47	83,01 ± 84,24	32,94 ± 42,73

Supplementary table 3: Protein filament structures from the electron microscopy data base compared to the ALM-associated filament. The filaments are listed by type. Supplementary Fig. 7 shows the subset of filaments being most relevant either by biological function or close structural match.

**class of filament**	**EMDB accession codes (EMD-)**
F-actin	11976, 6448
decorated F-actin	4346, 6446, 7831, 20711, 20843, 20844, 21155, 21925, 30085
CARD domain	6842, 7314, 8902, 8903, 9332, 9943, 9948, 22219, 22220
caspase	8300
CTP synthase	0840, 8474
DMC1	30311
Dvl2/DIX	21148
glucokinase	20309
IMPDH	4402, 8690
NLRP6	0438
MDA5-dsRNA	0143, 4338, 4341, 5444
MxB	8577
MyD88	4405
p62/SQSTM1	10499, 10500, 10501 10502
phosphofructokinase	8542
RAD51	8183, 9566
RIG-I-dsRNA	22371
Torsin	20076
VPS24	11212
