# Supplementary material for: Membrane-assisted assembly and selective secretory autophagy of enteroviruses
Source: Nat Commun. 2022 Oct 10;13:5986. doi: 10.1038/s41467-022-33483-7 (PMC9550805; doi:10.1038/s41467-022-33483-7)
Supplement: Supplementary file 3 — Description of Additional Supplementary Information [file 41467_2022_33483_MOESM3_ESM.pdf]

Description of Additional Supplementary Information

Supplementary Movie1: Tomographic volume of poliovirus induced replication complexes, corresponding to the region of the polio-infected cell milled at 6 h p.i. shown in Fig.1A.

Supplementary Movie2: Tomographic volume of poliovirus assembly on the replication membranes, corresponding to the region shown in Fig.3A of poliovirus-infected cell treated with MRT68921+ VPS34-IN1 and milled at 6h p.i.

Supplementary Movie 3: Tomographic volume of poliovirus-infected cell treated with MRT68921 shown in Fig.4A.

Supplementary Movie 4: Tomographic volume of the protein filament bundles trapped in phagophore-like membranes shown supplementary figure 6A.
