# Supplementary material for: Membrane-assisted assembly and selective secretory autophagy of enteroviruses
Source: Nat Commun. 2022 Oct 10;13:5986. doi: 10.1038/s41467-022-33483-7 (PMC9550805; doi:10.1038/s41467-022-33483-7)
Supplement: Supplementary file 8 — Reporting Summary [file 41467_2022_33483_MOESM8_ESM.pdf]

nature portfolio

Corresponding author(s): Lars-Anders Carlson

Last updated by author(s): 31/08/2022

Reporting Summary

Nature Portfolio wishes to improve the reproducibility of the work that we publish. This form provides structure for consistency and transparency in reporting. For further information on Nature Portfolio policies, see our Editorial Policies and the Editorial Policy Checklist.

Statistics

For all statistical analyses, confirm that the following items are present in the figure legend, table legend, main text, or Methods section.

n/a	Confirmed	
☐	☒	The exact sample size (*n*) for each experimental group/condition, given as a discrete number and unit of measurement
☐	☒	A statement on whether measurements were taken from distinct samples or whether the same sample was measured repeatedly
☐	☒	The statistical test(s) used AND whether they are one- or two-sided<br>*Only common tests should be described solely by name; describe more complex techniques in the Methods section.*
☐	☒	A description of all covariates tested
☐	☒	A description of any assumptions or corrections, such as tests of normality and adjustment for multiple comparisons
☐	☒	A full description of the statistical parameters including central tendency (e.g. means) or other basic estimates (e.g. regression coefficient) AND variation (e.g. standard deviation) or associated estimates of uncertainty (e.g. confidence intervals)
☐	☒	For null hypothesis testing, the test statistic (e.g. *F*, *t*, *r*) with confidence intervals, effect sizes, degrees of freedom and *P* value noted<br>*Give P values as exact values whenever suitable.*
☒	☐	For Bayesian analysis, information on the choice of priors and Markov chain Monte Carlo settings
☒	☐	For hierarchical and complex designs, identification of the appropriate level for tests and full reporting of outcomes
☒	☐	Estimates of effect sizes (e.g. Cohen's *d*, Pearson's *r*), indicating how they were calculated

Our web collection on statistics for biologists contains articles on many of the points above.

Software and code

Policy information about availability of computer code

Data collection	SerielEM 3.8
Data analysis	IMOD 4.11.6, CTFFIND4 4.1.8, MotionCor2 1.1.0, Dynamo 1.1.53, Pythom 2 .7.1.2, SubTom 1.1.6, GraphPad Prism 9.4.1, Amira 2022.1, chimerax 1.2.5, ImageJ 1.53c.

For manuscripts utilizing custom algorithms or software that are central to the research but not yet described in published literature, software must be made available to editors and reviewers. We strongly encourage code deposition in a community repository (e.g. GitHub). See the Nature Portfolio guidelines for submitting code & software for further information.

Data

Policy information about availability of data

All manuscripts must include a data availability statement. This statement should provide the following information, where applicable:

- Accession codes, unique identifiers, or web links for publicly available datasets
- A description of any restrictions on data availability
- For clinical datasets or third party data, please ensure that the statement adheres to our policy

The subtomogram average maps have been deposited in the Electron Microscopy Data Bank (EMDB) under accession codes EMD-15390 (empty capsid), EMD-15391 (RNA-loaded virion), EMD-15392 (tethered virion), and EMD-13682 (filament).

Human research participants

Policy information about studies involving human research participants and Sex and Gender in Research.

Reporting on sex and gender	n/a
Population characteristics	n/a
Recruitment	n/a
Ethics oversight	n/a

Note that full information on the approval of the study protocol must also be provided in the manuscript.

Field-specific reporting

Please select the one below that is the best fit for your research. If you are not sure, read the appropriate sections before making your selection.

- [x] Life sciences
- [ ] Behavioural & social sciences
- [ ] Ecological, evolutionary & environmental sciences

For a reference copy of the document with all sections, see nature.com/documents/nr-reporting-summary-flat.pdf

Life sciences study design

All studies must disclose on these points even when the disclosure is negative.

Sample size	No sample-size calculation was performed. The sample size was sufficient to interpret the data with statistical analysis and draw a biological conclusion.
Data exclusions	Subvolume classification of protein filaments resulted in 3D classes with interpretable filament structure and others without clear structure. Particles within those classes that did not align were discarded from further analysis.
Replication	The data here was collected over 17 independent sessions (more than 100 tomograms were generated) following independently reproducible replications of the experiments.
Randomization	Not applicable.
Blinding	Not applicable.

Reporting for specific materials, systems and methods

We require information from authors about some types of materials, experimental systems and methods used in many studies. Here, indicate whether each material, system or method listed is relevant to your study. If you are not sure if a list item applies to your research, read the appropriate section before selecting a response.

Materials & experimental systems

n/a	Involved in the study	
☐	☐	Antibodies
☐	☐	Eukaryotic cell lines
☒	☐	Palaeontology and archaeology
☒	☐	Animals and other organisms
☒	☐	Clinical data
☒	☐	Dual use research of concern

Methods

n/a	Involved in the study	
☒	☐	ChIP-seq
☒	☐	Flow cytometry
☒	☐	MRI-based neuroimaging

Antibodies

Antibodies used	Rabbit monoclonal anti-LC3B (D11, Cell Signaling), Mouse monoclonal anti-β-actin (A2228 - Sigma), Mouse monoclonal anti-VP1 (clone B3/H1), Rabbit polyclonal anti-3D gifted by George Belov (UMD – diluted 1:500), Human anti-A12 gifted by Konstantin Chumakov (FDA), Rabbit monoclonal anti-P62 (SQSTM1) (PM045, MBL). Goat anti-Rabbit IgG (H+L) Cross-Adsorbed Secondary Antibody, Alexa Fluor 555 (A21428, Invitrogen), Goat anti-Human IgG (H+L) Cross-Adsorbed Secondary Antibody, Alexa Fluor 488 (A11013, Invitrogen). HRP Goat Anti-Rabbit IgG H&L (ab205718) and HRP Mouse Rabbit Anti-Mouse IgG H&L (ab6728).

Validation	https://www.cellsignal.com/products/primary-antibodies/lc3b-d11-xp-rabbit-mab/3868<br>https://www.sigmaaldrich.com/SE/en/product/sigma/a2228<br>https://www.mblintl.com/products/pm045/<br>https://www.abcam.com/goat-rabbit-igg-hl-hrp-ab205718.html<br>https://www.abcam.com/rabbit-mouse-igg-hl-hrp-ab6728.html<br>https://www.thermofisher.com/antibody/product/Goat-anti-Rabbit-IgG-H-L-Cross-Adsorbed-Secondary-Antibody-Polyclonal/A-21428<br>https://www.thermofisher.com/antibody/product/Goat-anti-Human-IgG-H-L-Cross-Adsorbed-Secondary-Antibody-Polyclonal/A-11013<br><br>Poliovirus 3D antibody was produced and described earlier in this paper : Nchoutmboube J.A., Viktorova E.G., Scott A.J., Ford L.A., Pei Z., Watkins P.A., Ernst R.K., Belov G.A. Increased long chain acyl-Coa synthetase activity and fatty acid import is linked to membrane synthesis for development of picornavirus replication organelles. DOI: 10.1371/journal.ppat.1003401

Eukaryotic cell lines

Policy information about cell lines and Sex and Gender in Research

Cell line source(s)	HeLa cells were obtained from ATCC (# CRM-CCL-2™). HeLa LC3 and GABARAP 3KO cells were gifted by Michael Lazarou.
Authentication	HeLa cells were authenticated by ATCC. The KO cells were produced and described earlier in this paper: Padman et al, Autophagosome formation and cargo sequestration in the absence of LC3/GABARAPs. DOI: 10.1080/15548627.2017.1281492
Mycoplasma contamination	We regularly test our cell lines for mycoplasma. All of the used cells were negative.
Commonly misidentified lines (See ICLAC register)	Not applicable
